# Associations between U.S. Adult Obesity and State and County Economic Conditions in the Recession

**DOI:** 10.3390/jcm3010153

**Published:** 2014-01-27

**Authors:** Qi Zhang, Rajan Lamichhane, Youfa Wang

**Affiliations:** 1 School of Community and Environmental Health, Old Dominion University, 3138 Health Sciences Building, Norfolk, VA 23529, USA; 2 Department of Mathematics, Texas A & M University-Kingsville, Rhode Hall 231, MSC 172, Kingsville, TX 78363, USA; E-Mail: rajan.lamichhane@tamuk.edu; 3 Department of Social and Preventive Medicine, School of Public Health and Health Professions, University at Buffalo, State University of New York, Farber Hall, Room 270, Buffalo, NY 14214, USA; E-Mail: youfawan@buffalo.edu

**Keywords:** obesity, recession, unemployment rate, employment status

## Abstract

This study examines the association between state and county unemployment rates and individuals’ body weight status during the latest recession in the U.S. We used the U.S. Behavioral Risk Factor Surveillance System (BRFSS) data in 2007, 2009 and 2011, which were collected from 722,692 American adults aged 18 or older. Overweight and obesity were defined as body mass index (BMI) ≥25, and ≥30, respectively. Multivariate linear and logistic regressions were applied to assess the association between BMI, risks of overweight and obesity, and state and county unemployment rates. State unemployment rates were negatively associated with individual BMI across years, while county unemployment rates were significantly positively associated with BMI and obesity rates in all years (*p* < 0.05). However, the scale of the positive relationship was reduced in 2009 and 2011. Stratified analyses were conducted among adults with employment and without employment. The unemployed group’s body weight status was not related to state- and county-level economic conditions in most times. In the pooled analyses with all three years’ data, the relationship between unemployment rates and body weight status were consistently reduced after the recession of 2008–2009. Our results indicated that macroeconomic conditions at different levels can have different associations with individuals’ obesity risk across time.

## 1. Introduction

The scale, length and depth of the latest economic recession has exceeded all recessions since the Great Depression [1]. The U.S. economy reached its worst level in 26 years, while the national unemployment rate reached 9.3% in 2009, a 60.3% jump from the previous year [2]. This prolonged recession will further increase unemployment and poverty, potentially resulting in significant health consequences affecting millions of Americans [3,4]. 

The relationship between economic cycles and health outcomes has warranted continuous interest since the Great Depression in the 1930s. Earlier studies have demonstrated the negative impact of economic recessions on general health outcomes. Brenner demonstrated this association with time-series studies, observing increases in psychiatric hospital admission, infant mortality rates and deaths in recessions due to cardiovascular disease, cirrhosis, suicide and homicide [5,6,7]. Catalano and Dooley’s study found that an economic contraction negatively affects illness and injury rates by increasing the incidence of undesirable job and financial events for middle-income respondents only [8]. Likewise, Gerdtham and Johannsson found a highly significant effect of unemployment on mortality [9].

However, more recent evidence suggests that sudden or short-run economic upturns, instead of recessions, negatively affect health by creating more job stress, less time for self-care activities such as eating well or exercising, overindulgence in unhealthy food, and work-related accidents [10,11,12,13]. Ruhm [10] reported that state unemployment rates are significantly and negatively related to total mortality and to 8 out of 10 specific causes of fatality. A one percentage point rise in unemployment reduces the total death rate by 0.5% [10]. Ruhm also demonstrated that a rise in unemployment predicts reductions in the prevalence of medical problems, decline in acute morbidities and bed-days, and declines in ischemic heart disease and intervertebral disk disorder [14]. He clarified that while in the short-run, economic expansions are more likely to place stress on individuals, resulting in adverse health effects, permanent gains in economic improvements provide higher-level health through technological innovations, greater access to care and improved purchasing ability for items that provide greater safety, like newer automobiles [15]. 

Regarding economic recession and obesity, researchers have painted a mixed picture in the literature. Ruhm used the Behavioral Risk Factor Surveillance System (BRFSS) 1987–1995 data and found that during economic downturns people have more time to exercise and prepare healthy meals and are thus more likely to maintain a healthy weight [10]. Subsequently, he used data on adults from the BRFSS 1987–2000 to demonstrate that excess weight and physical inactivity decline when economic conditions improve [16]. Hruschka also used BRFSS data and compared the annual growth rates of BMI during 2004–2007 and 2008–2010 [17]. His results indicated that the annual change in BMI was significantly reduced across income groups after the 2008 recession. However, a Finnish study using individual microdata from 1978–2002 found that improvement in economic conditions produced a decrease in BMI [18]. Charles and DeCicca used data from the National Health Interview Survey (NHIS) during 1997–2001 and found that an increase in unemployment rates was associated with an increase in body weight status among those least likely to be employed (low-income and low-education) and African Americans [19]. The contrary findings in the literature suggest that further research is needed.

The most recent recession (2008–2009) was the severest since the Great Recession in the 1930s in terms of its length (18 months) [1]. Therefore, the recession in 2008–2009 provided a unique opportunity to examine the association between economic conditions and obesity. There are concerns that a major economic downturn could impose additional risks of obesity due to people having a lower dietary quality and engaging in less physical activity [20]. However, others have debated the actual health impact of the latest recession [21,22,23,24]. To provide more evidence to clarify the associations between economic conditions and obesity, we examined the associations between obesity and state or county unemployment rates using cross-sectional waves of the Behavioral Risk Factor Surveillance System (BRFSS) in 2007, 2009 and 2011, which encompass the periods before, during, and after the recession. 

## 2. Methods

### 2.1. Data

The BRFSS, a cross-sectional telephone survey of non-institutionalized American adults aged 18 years or older, has been an annual survey conducted by the Centers for Disease Control and Prevention (CDC) since 1984 that provides representative data at the national and state levels on health behaviors, preventive health practices and risk factors for the leading causes of death in the U.S. [25]. A multi-stage, disproportionate stratified sample (DSS) design was employed by the BRFSS consistently across years. After telephone numbers were randomly selected, computer-assisted telephone interviews were conducted by trained professionals in each state. After the interviews, the data were compiled at the CDC, which processed and prepared the national data for that year. More details about the BRFSS data collection are available [25]. Three waves of BRFSS data, 2007, 2009 and 2011, were used in this study. The national unemployment rate in 2007 was 4.6%, while the numbers in 2009 and 2011 were 9.3% and 8.9%, respectively, so these three years captured the window period before and after the recession of 2008–2009. We linked publicly available BRFSS data with Local Area Unemployment Statistics (LAUS) for state and county unemployment rates [2]. 

### 2.2. Measurement

Outcome variables: Body mass index (BMI) was defined as weight (kg)/height^2^ (m^2^); overweight if BMI ≥25; and obesity if BMI ≥30.

Key exposure variables: State and county unemployment rates. 

Covariates: Socio-demographic variables, such as employment status, race/ethnicity, education, age, and income group, were controlled. Employment status in the BRFSS was classified as “employed for wages”, “self-employed”, “out of work for more than 1 year”, “out of work for less than 1 year”, “a homemaker”, a “student” and “retired”. In our analyses, the “employed” groups included adults who were employed for wages or self-employed; the “unemployed” groups included adults who were “out of work for more than 1 year” and “out of work for less than 1 year”. Since the retired group was no longer associated with labor market outcomes, we removed them from the analyses. Since the unemployment rates can affect both employed and unemployed adults’ lifestyles, we stratified the analyses for the employed and the unemployed groups. Race/Ethnicity was categorized as “non-Hispanic whites”, “non-Hispanic blacks”, “Hispanics” and “Other”. Education was classified as “<high school”, “high school”, “some college”, and “≥college graduate”. Income groups were created as “<$15,000”, “$15,000–$25,000”, “$25,000–$35,000”, “$35,000–$50,000” and “≥$50,000”. We also controlled the state per capita Gross Domestic Product (GDP) as the proxy for state-level economic indicators, since the GDP may fluctuate in business cycles. For other unobserved heterogeneity at the state level, we used dummies as a control in the regressions. Smoking behavior, current smoker or not, was also added as a control for health behavior and an indicator for general health. 

### 2.3. Statistical Analysis

Our analysis was conducted using Stata, Version 11 (Stata Press, College Station, TX, USA) and took into account the complex survey design of the BRFSS. First, we calculated the descriptive statistics of the study samples from the BRFSS in each year. Next, we conducted multivariate linear and logistic regression to examine the associations between BMI, overweight and obesity with state or county unemployment rates across years. Since the BRFSS is a cross-sectional survey, we are unable to conduct a longitudinal study to examine how the unemployment changes were associated with changes in individual body weight status. To address this limitation partially, we pooled the data from 2007, 2009 and 2011 and added the interactive terms of unemployment and survey years to test whether the relationship between the unemployment rates and body weight status changed across the two years. Due to the high correlation between state and county unemployment rates, we conducted the regression separately for state- and county-level unemployment rates. 

## 3. Results

Table 1 presents the summary statistics of the subjects interviewed in the BRFSS 2007 to 2011 and the pooled sample of the three years. Our analysis sample included individuals with a mean age of 41.96 years in the pooled sample. Approximately half of them were men; 66.24% of them were non-Hispanic white, 10.88% were non-Hispanic black, 16.89% were Hispanics. The unemployment rates were 5.22% in 2007, 9.84% in 2009, and 10.28% in the pooled sample. The almost doubled unemployment rates after 2008 reflected the severity of the latest recession. Although the recession officially ended in June 2009 based on the National Bureau of Economic Research, labor market conditions were not immediately improved. The proportions of homemakers were essentially the same, while greater proportions of the surveyed individuals were students (4.63% in 2007, 5.00% in 2009, and 5.83% in 2011). 

**Table 1:** Descriptive Statistics of the Behavioral Risk Factor Surveillance System (BRFSS) 2007, 2009 and 2011.

	2007		2009		2011		Pooled (2007, 2009 & 2011)	
	Mean or %	Standard error	Mean or %	Standard error	Mean or %	Standard error	Mean or %	Standard error
*N*	224,671		234,534		263,487		722,692	
Age (years)	41.52	0.07	41.95	0.06	42.38	0.08	41.96	0.04
Men (%)	50.49	0.24	50.51	0.21	50.91	0.198	50.64	0.126
**Race/Ethnicity**								
Non-Hispanic White (%)	66.53	0.30	67.05	0.24	65.13	0.37	66.24	0.17
Non-Hispanic Black (%)	10.24	0.15	10.47	0.15	11.91	0.19	10.88	0.09
Hispanics (%)	16.96	0.28	16.78	0.21	16.95	0.30	16.89	0.14
Other (%)	6.28	0.15	5.70	0.12	6.01	0.14	5.98	0.08
**Employment Status (%)**								
Employed	75.73	0.21	70.86	0.20	68.93	0.21	71.72	0.12
Unemployed	5.22	0.12	9.84	0.14	10.28	0.13	8.56	0.08
Homemaker	8.84	0.12	8.33	0.10	7.37	0.10	8.16	0.06
Students	4.63	0.14	5.00	0.13	5.83	0.12	5.17	0.07

Table 2 presents the results from the linear regression between BMI and the state and county unemployment rates across years. Each cell in Table 2 represents one regression result. In all years for the total group, state unemployment rates had a negative relationship with BMI, although the coefficient was not significant in 2009. However, the signs of these coefficients varied by gender and employment groups. Among men, the state unemployment rate was not a significant factor associated with BMI in all years, although the results were significant among women. Among the employed adults, the state unemployment rates were negatively associated with BMI and the coefficients were significant in 2007 and 2011. For unemployed adults, the signs of the coefficients were mixed for state unemployment rates. The coefficients in 2009 and 2011 were positive, while the coefficient in 2007 was negative. 

County unemployment had a more consistent association with BMI. In all years, the coefficients were all positive in all gender and employment groups, except the unemployment groups in 2007 (beta = −0.03, *p* > 0.05). The positive associations indicate that on average, individuals living in counties with higher unemployment rates had a greater BMI and the relationship was statistically significant in all groups except for the unemployed groups. For all the significant groups, the scale of the coefficients was much smaller in 2009 and 2011 than in 2007, e.g., the total group’s beta of county unemployment was reduced to 0.10 in 2009 and 2011, down from 0.19 in 2007. The same patterns were found in men, women and the employed groups, which indicate that the associations between county unemployment rates and individual BMIs were weakened by the economic recession in 2008, although the county unemployment rates remained a significant predictor.

**Table 2:** Linear relationship between body mass index and state and county unemployment (unemp) rates among BRFSS participants in 2007, 2009 and 2011.

	Body Mass Index								
	2007			2009			2011		
	Beta	Standard error	*p*-Value	Beta	Standard error	*p*-Value	Beta	Standard error	*p*-Value
**Total**									
State Unemp Rate	−0.37	0.13	**	−0.27	0.21		−1.53	0.67	*
County Unemp Rate	0.19	0.03	***	0.10	0.01	***	0.10	0.01	***
**By sex**									
**Male**									
State Unemp Rate	−0.03	0.19		0.26	0.32		0.74	0.24	
County Unemp Rate	0.15	0.05	**	0.10	0.02	***	0.09	0.02	***
**Female**									
State Unemp Rate	−0.74	0.20	***	−0.85	0.27	**	−3.00	0.83	***
County Unemp Rate	0.23	0.04	***	0.08	0.02	***	0.12	0.02	***
**By Employment** **Status**									
**Employed**									
State Unemp Rate	−0.31	0.14	*	−0.22	0.22		−1.97	0.68	**
County Unemp Rate	0.21	0.03	***	0.10	0.02	***	0.11	0.01	***
**Unemployed**									
State Unemp Rate	−0.23	0.96		2.25	1.11	*	3.55	2.86	
County Unemp Rate	−0.03	0.12		0.01	0.04		0.09	0.04	*

*** <0.001; ** <0.01; * <0.05; Models controlled for age, gender, race/ethnicity, income, education, state level GDP per capita, smoking behavior, state dummies and employment status when the analysis was not conducted; Overweight: BMI ≥25; Obesity: BMI ≥30.

The associations between unemployment rates and risks of overweight were similar in Table 3 as the results in Table 2. Adults in states with higher unemployment rates were less likely to be overweight, especially for women and employed adults. Among men and unemployed group, there was no significant relationship between state unemployment rates and overweight. The odds ratios (ORs) for county unemployment rates were consistently positive, but they were not significant among men in 2007 and unemployed groups in 2007 and 2009. Table 4 presents the results with obesity as the outcome. The signs of state unemployment rates were more mixed than in Table 2 and Table 3, while the significance levels were reduced compared with the results in Table 2 and Table 3. In general, state unemployment rates were not significantly associated with obesity, except among women. In 2007 and 2009, women in states with higher unemployment rates were significantly less likely to be obese, while this relationship became insignificant in 2011. County unemployment rates remained a significantly positive risk factor for obesity in all groups, except the unemployed adults. However, the scales of the ORs were clearly reduced in 2009 and 2011 compared with those in 2007. 

**Table 3:** Individual risk of overweight among BRFSS participants in 2007, 2009 and 2011 by state and county unemployment rates.

	Overweight								
	2007			2009			2011		
	OR	*p*-Value	95% CI	OR	*p*-Value	95% CI	OR	*p*-Value	95% CI
**Total**									
State Unemp Rate	0.87	*	0.78–0.97	0.97		0.83–1.13	0.52	**	0.34–0.78
County Unemp Rate	1.06	***	1.03–1.08	1.04	***	1.03–1.05	1.04	***	1.03–1.05
**By sex**									
**Male**									
State Unemp Rate	1.01		0.84–1.20	1.06		0.82–1.39	0.74		0.39–1.41
County Unemp Rate	1.04		1.00–1.08	1.05	***	1.03–1.06	1.04	***	1.03–1.06
**Female**									
State Unemp Rate	0.76	***	0.67–0.88	0.88		0.74–1.06	0.40	**	0.23–0.71
County Unemp Rate	1.07	***	1.05–1.10	1.04	***	1.02–1.05	1.03	***	1.02–1.05
**By Employment Status**									
**Employed**									
State Unemp Rate	0.87	*	0.80–0.98	1.00		0.84–1.19	0.49	**	0.29–0.83
County Unemp Rate	1.06	***	1.03–1.09	1.04	***	1.03–1.05	1.04	***	1.03–1.05
**Unemployed**									
State Unemp Rate	1.35		0.75–2.43	1.42		0.77–2.64	1.06		0.28–9.15
County Unemp Rate	1.03		0.93–1.14	1.01		0.99–1.05	1.06	***	1.03–1.10

*** <0.001; ** <0.01; * <0.05; Models controlled for age, gender, race/ethnicity, income, education, state level GDP per capita, smoking behavior, state dummies and employment status when the analysis was not conducted; Overweight: BMI ≥25; Obesity: BMI ≥30.

**Table 4:** Individual risk of obesity among BRFSS participants in 2007, 2009 and 2011 by state and county unemployment rates.

	Obesity								
	2007			2009			2011		
	OR	*p*-Value	95% CI	OR	*p*-Value	95% CI	OR	*p*-Value	95% CI
**Total**									
State Unemp Rate	0.93		0.84–1.03	0.98		0.85–1.13	0.63		0.39–1.04
County Unemp Rate	1.07	***	1.04–1.09	1.03	***	1.02–1.04	1.04	***	1.02–1.05
**By sex**									
**Male**									
State Unemp Rate	1.02		0.87–1.19	1.14		0.92–1.42	0.93		0.46–1.89
County Unemp Rate	1.07	***	1.03–1.11	1.04	***	1.02–1.05	1.04	***	1.02–1.05
**Female**									
State Unemp Rate	0.84	**	0.73–0.96	0.82	*	0.68–0.99	0.42		0.21–0.84
County Unemp Rate	1.07	***	1.04–1.1	1.03	***	1.02–1.04	1.03	***	1.02–1.05
**By Employment Status**									
**Employed**									
State Unemp Rate	0.93		0.83–1.05	0.97		0.83–1.14	0.51	*	0.29–0.88
County Unemp Rate	1.07	***	1.04–1.1	1.04	***	1.02–1.05	1.04	***	1.03–1.05
**Unemployed**									
State Unemp Rate	1.55		0.9–2.68	1.85	*	1.03–3.3	2.21		0.36–13.45
County Unemp Rate	0.97		0.89–1.06	1.02		0.99–1.05	1.04	*	1.02–1.08

*** <0.001; ** <0.01; * <0.05; Models controlled for age, gender, race/ethnicity, income, education, state level GDP per capita, smoking behavior, state dummies and employment status when the analysis was not conducted; Obesity: BMI ≥30.

To test formally whether the association between unemployment rates and body weight status changed across three waves, we pooled the data of 2007, 2009 and 2011. The interactive terms were added in the analyses, while 2009 was used as the reference year. The results are presented in Table 5. For total groups, the interactive terms of state unemployment rates with year 2007 was negative for BMI, overweight and obesity, while the interactive term was significant for BMI. However, the interactive terms between state unemployment rates and year 2011 were highly significant for all three definitions of body weight status (*p* < 0.001), which means the relationship between unemployment rates and obesity was significantly reduced after the recession. The interactive terms between county unemployment rates and year 2007 were all significantly positive for all body weight statuses (beta > 0, and OR >1), and those of year 2011 were all significantly negative (beta < 0 and OR < 1). Therefore, the relationship between county unemployment rates and obesity was stronger prior to the recession, while weaker after the recession. Similar patterns were found for men, women and the employed group, with the unemployed group as an exception. Among the unemployed group, none of the interactive terms were significant, which indicates the relationship between unemployment rates and body weight status did not change during the economic recession. 

**Table 5:** Pooled analysis of relationship between body mass index, body weight status and state- and county unemployment rates in the BRFSS adults in 2007, 2009 and 2011 by participant characteristics.

	Body Mass Index			Overweight			Obesity		
	Beta	SE	*p*-Value	OR	*p*-Value	95% CI	OR	*p*-Value	95% CI
**Total**									
State Unemp Rate	0.01	0.02		1.01		0.99–1.02	1.00		0.99–1.02
State Unemp Rate (Year 07)	−0.04	0.02	*	0.99		0.98–1.01	0.99		0.97–1.00
State Unemp Rate (Year 11)	−0.01	0.00	***	0.99	***	0.98–0.99	0.99	***	0.99–0.997
County Unemp Rate	0.10	0.01	***	1.04	***	1.03–1.04	1.04	***	1.03–1.04
County Unemp Rate (Year 07)	0.04	0.01	***	1.02	***	1.01–1.03	1.01	***	1.01–1.02
County Unemp Rate (Year 11)	−0.01	0.004	**	0.99	***	0.98–0.99	0.99	***	0.99–0.997
**By sex**									
**Male**									
State Unemp Rate	0.001	0.03		1.01		0.99–1.04	1.00		0.98–1.03
State Unemp Rate (Year 07)	−0.05	0.03		1.00		0.98–1.02	0.99		0.96–1.01
State Unemp Rate (Year 11)	−0.02	0.01	**	0.99	*	0.98–0.99	0.99	**	0.98–0.99
County Unemp Rate	0.08	0.01	***	1.04	***	1.03–1.05	1.04	***	1.03–1.05
County Unemp Rate (Year 07)	0.05	0.01	**	1.02	*	1.00–1.03	1.01	*	1.00–1.03
County Unemp Rate (Year 11)	−0.01	0.01	*	0.99	*	0.98–0.99	0.99	**	0.98–0.997
**Female**									
State Unemp Rate	0.02	0.03		1.00		0.98–1.02	1.01		0.99–1.03
State Unemp Rate (Year 07)	−0.04	0.03		0.99		0.97–1.00	0.99		0.97–1.01
State Unemp Rate (Year 11)	−0.01	0.01		0.99	**	0.98–0.99	0.99	*	0.99–0.999
County Unemp Rate	0.10	0.01	***	1.04	***	1.03–1.04	1.03	***	1.02–1.04
County Unemp Rate (Year 07)	0.05	0.01	***	1.02	***	1.01–1.03	1.01	**	1.01–1.02
County Unemp Rate (Year 11)	−0.004	0.01		0.99	**	0.99–0.998	0.99	*	0.99–0.999
**By Employment Status**									
**Employed**									
State Unemp Rate	0.01	0.02		1.01		0.99–1.03	1.00		0.98–1.02
State Unemp Rate (Year 07)	−0.05	0.02	*	1.00		0.98–1.01	0.98		0.97–1.00
State Unemp Rate (Year 11)	−0.02	0.004	***	0.99	***	0.99–0.996	0.99	***	0.98–0.99
County Unemp Rate	0.11	0.01	***	1.04	***	1.03–1.05	1.04	***	1.03–1.05
County Unemp Rate (Year 07)	0.04	0.01	***	1.02	**	1.01–1.03	1.02	**	1.01–1.02
County Unemp Rate (Year 11)	−0.01	0.004	**	0.99	***	0.99–0.997	0.99	***	0.99–0.996
**Unemployed**									
State Unemp Rate	−0.05	0.10		1.01		0.95–1.07	0.99		0.94–1.06
State Unemp Rate (Year 07)	−0.10	0.09		0.99		0.93–1.05	0.99		0.93–1.04
State Unemp Rate (Year 11)	−0.01	0.01		0.99		0.98–1.01	0.99		0.99–1.01
County Unemp Rate	0.03	0.03		1.04	***	1.02–1.06	1.02	*	1.00–1.04
County Unemp Rate (Year 07)	−0.02	0.04		1.01		0.98–1.04	1.00		0.98–1.03
County Unemp Rate (Year 11)	−0.004	0.01		0.99		0.99–1.00	1.00		0.99–1.01

*** <0.001; ** <0.01; * <0.05; Models controlled for age, gender, race/ethnicity, income, education, state level GDP per capita, smoking behavior, state dummies and employment status when the analysis was not conducted; Overweight: BMI ≥25; Obesity: BMI ≥30.

In summary, comparing the associations of unemployment rates with individual body weight status during, before and after the economic recession of 2008–2009, we found the following patterns: First, the positive relationship between the county unemployment rates and body weight status was reduced after the economic recession; Second, the reduction of the relationship between state unemployment rates and body weight status after the recession was more evident among women and the employed adults; Lastly, little significant relationship was found among the unemployed adults, and this pattern did not change after the recession. 

## 4. Discussion

Using three waves of the latest nationally representative data, our study suggests that the recent economic recession was associated with American adults’ body weight status. The association was different at the state and the county levels and changed in the periods before, during, and after the latest recession of 2008–2009. 

We found that the negative association between state unemployment and individual body weight status, especially among women and employed adults. These findings echo previous research about the health benefits of recession if the economic condition is indicated by the state unemployment rate [10,16]. At the county level, higher unemployment rates were still associated with higher BMI and obesity risks. However, the scale of this association was reduced as well in the recession, which is consistent with the latest finding that the annual growth rates of body weight slowed after the recession [17]. The opposite directions of economic conditions and obesity at different levels (state and county) reflect the recent debate on the health outcomes of the current recession [19]. Our results indicate that the state economic condition may not bring a significant shock to an individual’s body weight status, but worse local economic conditions can be a significant risk factor for obesity, especially in a booming time [18]. 

In boom times or in recession, there was little significant relationship between state or county unemployment and unemployed adults’ body weight status. There has been no clear answer in the literature to explain why unemployed adults’ body weight status is not associated with macroeconomic conditions. One possible explanation is that the detrimental health effect of unemployment alone is dominant, so that macroeconomic conditions may not have an additional impact on unemployed individuals’ health [26]. Another possibility is that unemployed adults may rely on federal assistance programs, such as the Supplemental Nutrition Assistance Program (SNAP), which does not vary significantly across regions. With the massive levels of unemployment in the severe recession in 2008–2009, the unemployed might thus be more homogeneous in dietary intake and physical activity than employed adults, regardless of local- or state-level economic conditions [16,27]. Since regional economic condition is little associated with obesity risks of the unemployed, standard programs across the nation can be implemented to promote a healthy lifestyle among adults without employment. One caveat of our study is that we did not examine change in employment status and its association with obesity risk, although the literature suggests that changes in individual employment status across business cycles may cause changes in lifestyles and health afterwards [10,15,18]. Researchers in the future may use more longitudinal data to understand fully the changes in health status among unemployed adults across business cycles. 

On the other hand, employed adults’ body weight status was more likely to be associated with state and county unemployment rates. They were more likely to be obese in counties with higher unemployment rates. Literature has documented the causal effect of economic recession and its detrimental effect on mental health, including stress [28]. Biological studies have indicated that chronic stress may induce human beings to increase their food intake as a comforting mechanism [29]. Therefore, employed adults in counties with high unemployment rates may work longer hours and/or experience more stress due to greater job insecurity. However, the latest recession of 2008–2009 was so severe that the economic conditions may have become quite miserable in most counties, which might have leveled the association between county unemployment rates and individuals’ obesity risk. That explains why the scale of the relationship at the county level was reduced in or after the recession compared with that in 2007. 

A few limitations should be acknowledged: First, the BRFSS collects self-reported body weights and heights based upon telephone surveys, which suffer reporting bias. Literature suggests that women are more likely to underreport their weight, while men are more likely to over-report their height [30]. Therefore, the interpretation of our results should be taken with caution due to the possible self-reporting bias in the original BRFSS data; Second, the BRFSS is a cross-sectional survey, so we were unable to track the same individuals across time and observe their changes in body weight status; Third, our study is one of the first studies that examine the latest economic recession and its health impact with regard to obesity. However, we only focused on the association instead of the causation between unemployment rates and body weight status. A longitudinal study with panel data may fully establish causality between economic recessions and individual health risks. Finally, the mechanism of how macroeconomic conditions affect individuals’ health status is complex and understudied, *i.e.*, how the regional economic shocks get “under the skin” is still an unknown. The seemingly contradictory findings at the state and county levels could be also due to the heterogeneity in other unobserved factors. This line of research is beyond the question solely of economics, and more multidisciplinary research is desirable. 

## 5. Conclusions

In summary, our study provided a preliminary look at the association between state- and county-level unemployment rates and adults’ body weight status during one of the most severe recessions since the Great Depressions in the 1930s. Our results indicate that the recession did not bring significant increases in obesity risk across gender and employment groups, which provides important evidence in the debate on the health impact of the latest recession.
